# Hiseq Base Molecular Characterization of Soil Microbial Community, Diversity Structure, and Predictive Functional Profiling in Continuous Cucumber Planted Soil Affected by Diverse Cropping Systems in an Intensive Greenhouse Region of Northern China

**DOI:** 10.3390/ijms20112619

**Published:** 2019-05-28

**Authors:** Ahmad Ali, Muhammad Imran Ghani, Yuhong Li, Haiyan Ding, Huanwen Meng, Zhihui Cheng

**Affiliations:** College of Horticulture, Northwest A&F University, Yangling 712100, China; ahmadhort87@nwafu.edu.cn (A.A.); imran_pak@nwsuaf.edu.cn (M.I.G.); liyuhong73@nwsuaf.edu.cn (Y.L.); woaimama195710@nwsuaf.edu.cn (H.D.); menghw2005@nwsuaf.edu.cn (H.M.)

**Keywords:** cucumber double cropping, winter catch cover crops, soil quality, microbial community, high-throughput sequencing, 16S rRNA gene

## Abstract

Cover crops are key determinants of the ecological stability and sustainability of continuous cropping soils. However, their agro-ecological role in differentially reshaping the microbiome structure and functioning under a degraded agroecosystem remains poorly investigated. Therefore, structural and metabolic changes in soil bacterial community composition in response to diverse plant species were assessed. Winter catch leafy vegetables crops were introduced as cover plants in a cucumber-fallow period. The results indicate that cover crop diversification promoted beneficial changes in soil chemical and biological attributes, which increased crop yields in a cucumber double-cropping system. Illumina high-throughput sequencing of *16S rRNA* genes indicated that the bacterial community composition and diversity changed through changes in the soil properties. Principal component analysis (PCA) coupled with non-metric multidimensional scaling (NMDS) analysis reveals that the cover planting shaped the soil microbiome more than the fallow planting (FC). Among different cropping systems, spinach–cucumber (SC) and non-heading Chinese cabbage–cucumber (NCCC) planting systems greatly induced higher soil nutrient function, biological activity, and bacterial diversity, thus resulting in higher cucumber yield. Quantitative analysis of linear discriminant analysis effect size (LEfSe) indicated that *Proteobacteria, Actinobacteria, Bacteroidetes, and Acidobacteria* were the potentially functional and active soil microbial taxa. Rhizospheres of NCCC, leaf lettuce–cucumber (LLC), coriander–cucumber (CC), and SC planting systems created hotspots for metabolic capabilities of abundant functional genes, compared to FC. In addition, the predictive metabolic characteristics (metabolism and detoxification) associated with host–plant symbiosis could be an important ecological signal that provides direct evidence of mediation of soil structure stability. Interestingly, the plant density of non–heading Chinese cabbage and spinach species was capable of reducing the adverse effect of arsenic (As) accumulation by increasing the function of the arsenate reductase pathway. Redundancy analysis (RDA) indicated that the relative abundance of the core microbiome can be directly and indirectly influenced by certain environmental determinants. These short-term findings stress the importance of studying cover cropping systems as an efficient biological tool to protect the ecological environment. Therefore, we can speculate that leafy crop diversification is socially acceptable, economically justifiable, and ecologically adaptable to meet the urgent demand for intensive cropping systems to promote positive feedback between crop–soil sustainable intensification.

## 1. Introduction

The interacting effect of agricultural management practices and cropping systems can evidently influence plant biodiversity, soil health, and productivity by triggering soil biochemical modification and soil microbial activity and composition [1,2,3]. However, the modern agricultural system is often intensified by single repetitions of crop species, characterized as monoculture [4,5,6]. Consecutive monoculture systems driven by a long-term anthropogenic influx of agrochemicals leads to serious replanting problems that severely induce phenomena known as continuous cropping obstacles (CCOs) [7]. Soil sickness, autotoxicity, nutrient imbalance, and deterioration of soil quality have been reported as the main causes of CCOs in intensive production systems [7,8,9]. Soil microorganisms drive important soil biogeochemical processes and are the key drivers of organic matter (OM) turnover and soil nutrient cycling in agricultural ecosystems. The components of soil microbial communities (SMCs) and diversity are imperative to maintain plant biodiversity, soil health, and productivity [10,11]. Empirical studies have documented that disturbance of the soil microbial ecosystem is thought to be one of the main mechanisms induced by CCOs, playing an elemental role in exhausting the soil biodiversity in many different ecosystems. Rhizosphere soil is considered the most sensitive to land-management practices, such as cropping systems, irrigation, and fertilization, compared to bulk soil [12]. The numbers of active microorganisms, including plant pathogens, plant-beneficial microorganisms, and saprotrophs, might also affect the activity and diversity of soil microbial communities [1]. Therefore, these drastic soil management practices may have a direct effect on soil microbial communities by eliminating soil biological activity [7,8,13].

Northern plastic greenhouse vegetable cropping (PGVC) is one of the most intensified areas of horticultural crops, contributing over 33% of total PGVC consumption demand across mainland production [5,14]. For example, a cucumber double-cropping system is a typical intensive production model with winter–spring (WS) and autumn–winter (AW) seasons under the PGVC structure. Consecutive monoculture is a core cropping system that has been accelerated by long-term anthropogenic inputs and manipulative treatments, resulting in substantial nutrient leaching, groundwater contamination, salinization, or acidification of commercial greenhouse vegetable soils [15,16,17]. Previous monoculture studies reported that cucumber cultivation with seven years of anthropogenic management practices caused reductions of 50% of plant biomass [5,6], and 31–42% of soil organic matter [18,19] and serious Fusarium wilt [8]. Further, high-throughput sequencing indicated that current soil management practices and conventional cropping systems for this type of cultivation are not eco–physiologically sound and the soil microbiome structure is greatly altered by continuous monocropping of cucumber [20]. Little is known about how specific microbial taxa are affected by such radical changes.

In this framework, traditional agroecologcial practices that allow degraded soils to “rest” or lie fallow during main cropping cycles are used throughout the world to improve the soil environment [21]. Traditional fallows often improve degraded soils only slightly, however, and some negative soil–plant consequences might be associated with fallow seasons. For example, [22] and [23] reported that the summer fallow season during cucumber growth can lead to depleted soil nutrients and impaired soil functions, and may cause groundwater pollution and soil-borne pathogens. Regional adaptive practices with technological improvements in crop production and soil health management, now or in the future, are pragmatic options for transforming to ecologically sustainable agricultural practices. Recognizing that traditional monoculture systems could have destructive consequences for cash vegetable production, one alternative to traditional fallows is to grow cover crops during the fallow period. A number of cover crops have been incorporated for the dual benefits of soil quality and crop yield [24,25]. However, not all cover crop diversification systems can result in soil-plant improvmennts. An increasing number of studies have suggested that the particular planting species during fallow growth of succeeding crops can have a significant impact on the soil biota structure by promoting ecological sustainability [1,26,27], where monoculture is primarily conceived as a regular practice at both the field and regional scale. In this context, embedding diverse planting systems may show a concomitant increase in agroecosystem function in terms of multiple environmental and economic benefits [28]. It has been posited that management practices such as crop rotation or multicropping, by increasing above-ground biodiversity, can result in corresponding increases in diversity below the ground [29].

Cover crop diversification in conventional cropping system is perceived to be ecologically intensive and biologically diversified to balance productivity, profitability, and environmental health [30,31]. Cover crop species with compatible niches tend to have more productive feedback during fallow periods in terms of soil structure stability, higher organic matter input, carbon sequestration, nutrient recycling, and disease suppression [32]. The widespread adoption of such dynamic practices suggests a greater abundance and diversity of key microbial decomposers [33]. Such short-term impact through cover crops can contribute to soil fertility in areas associated with long-term negative plant-soil feed-backs due to CCOs. A few studies have shown the interplay of cover crop and soil microbial community structure behind this positive effect. Less well known is whether above-ground crop diversity is coupled with below-ground microbial biodiversity and how this affects function with leafy vegetable diversification in intensive greenhouse production [34]. Specifically, comparative metagenomic understanding of the temporal variability and forces driving rhizosphere bacterial communities in different leafy vegetable-based cover plantations are limited, and cash vegetable crops under PGVC conditions remain to be studied. In addition, coherent empirical work has shown the ecological significance of rhizosphere communities of cucumber continuously planted in soil that is preferentially colonized by Proteobacteria, Bacteroidetes, and Actinobacteria under diversified cropping models [35,36]. The molecular characterization of these biomarkers is important to determine the metabolic capabilities of specific microbial groups in each niche, for example, with high-throughput 16S rDNA sequencing and microarray-based metagenomic approaches, such as pathway enrichment analysis by Tax4Fun or PICRUSt tools, to quantify the hundreds and thousands of functional genes from known microbes in each sample [37]. However, little is known about which microorganisms are active in the rhizosphere and what functional genes and pathways are present. Such studies bridge the gap between microbial diversity and predictive functional capabilities, which have been previously neglected or separately investigated for the rhizosphere flux of all agriculture systems [1].

Here, we selected leafy vegetables as the model plant species, grown during a fallow period in degraded, continuously planted soil. Their cover cropping effect was expected to be stronger for double-cropped cucumber due to the diverse crop density and biomass input. We speculated that rhizosphere soil microbial abundance, community composition, and functional diversity would change after incorporating different planting systems. High-throughput sequencing (Illumina HiSeq) was employed to analyze the microbial community structure and diversity under different cropping systems. The objectives were to (1) identify the appropriate cover cropping system to maintain soil quality, (2) determine the appropriate agricultural cropping system to induce the soil microbial community, and (3) identify the active microbiome and predict its functional capabilities under different cropping systems. The results of this work will provide a foundation for regulating soil quality profiling and microorganism community structure, guiding cropping system decisions, and protecting soil ecology

## 2. Results

### 2.1. Soil Properties and Cucumber Yield

The influences of different winter catch cropping systems on soil properties and cucumber yield in the WS and AW seasons are given in Table 1 and Table 2. The differences due to cover cropping effect on soil characteristics indicate that soil pH was slightly affected (*p* < 0.05) in both seasons but the values remain non-significant under all cropping systems. The electrical conductivity (EC) level changed greatly and a significant difference (highest EC level) was observed under both seasons of SC treatment cropping system more than the fallow–cucumber (FC) system. Spinach–cucumber (SC) and coriander–cucumber (CC) cropping systems exhibited higher soil OM content in the WS season compared with FC, and they consumed greater quantities of available P and K, respectively, in the WS season than the FC system (Table 1). Meanwhile, the non-heading Chinese cabbage–cucumber (NCCC) cropping system obviously improved the soil available nutrient contents in the next growth period of the AW season (Table 2). With regard to soil biological function, the CC cropping system during WS and AW seasons was highly conducive of invertase activity. The SC cropping system was efficient where soil urease, catalase, and alkaline phosphatase activity was documented as being higher in the WS season when compared with that in the FC cropping system (Table 1). These biological indicators were 6.90, 10.90, and 25.68 mg g^−1^, respectively, which were evidently higher under the NCCC cropping treatment than FC during the AW season (Table 2).

The seasonal cucumber yield was also affected under different cropping systems from WS to AW season (Table 3). Mean cucumber yield (55.23 kg/plot) for the SC cropping system was 19.5% greater than that for the FC system, and yield differences were significant (*p* < 0.05) in the WS season of 2017. Similarly, we found that the mean cucumber yield (6.16 kg/plot) for the NCCC planting system was 45.2% higher than that for the FC system, and the yield differences were significant (*p* < 0.05) in the AW season of 2017. By contrast, we found that mean production yield was 16% lower in the leafy lettuce–cucumber (LLC) plots in the AW season than the FC system.

### 2.2. Taxonomic Characterization of Rhizosphere Microbiota

After amplifying the V3–V4 region of the bacterial *16S rRNA* gene, the structure and composition of the community were analyzed by high-throughput sequencing. For the entire sampling set (15 soil replicates), a total of 951,364 sequences (raw tags) with an average length of 436 bp were identified using Illumina HiSeq analysis. As a result of chimeral filtration and quality control, 949,508 high-quality sequences (clean tags) were obtained. Finally, a total of 901,051 processed sequences (effective tags) were found in all samples, accounting for 94.7% of the total quantified sequences. The number of quality reads in soil samples was in the range of 53,462 to 69,760 in the 15 replicated treatments (Supplementary Table S1). These classified sequences were further used to cluster operational taxonomic units (OTUs) at a 3% dissimilarity level, and a total of 23,872 OTUs were netted, and species taxonomic analysis was also computed (Supplementary Table S2). All the sampling efforts tended to reach the saturation plateau in rarefaction analysis (Supplementary Figure S1) and were effective in covering the full extent of almost a majority of bacterial diversity at 97% sequence similarity in the rank abundance curve approach (Supplementary Figure S2).

### 2.3. Changes in Bacterial Community Composition and Diversity

**Figure 1:**
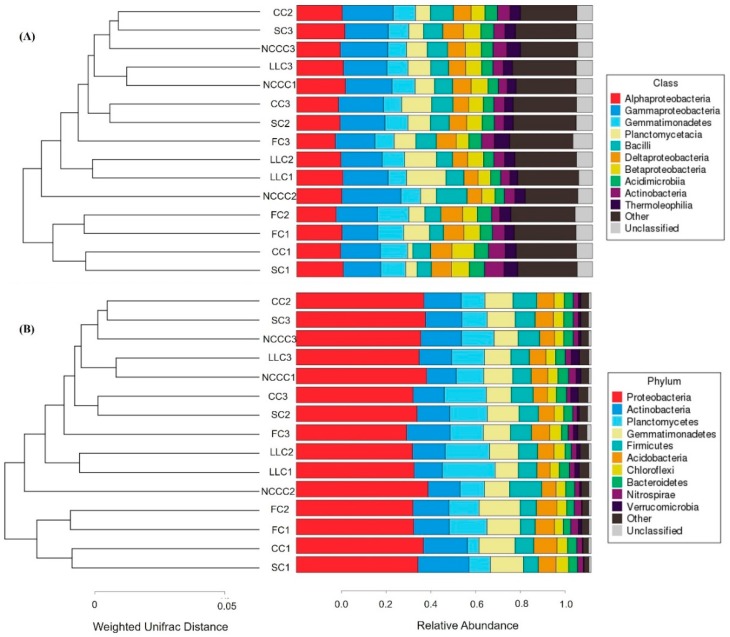
UniFrac UPGMA (unweighted pair group method with arithmetic mean) clustering analysis revealed the dominant soil bacterial class (**A**) and phyla (**B**) in all soil samples under different treatments. Relative abundances % of bacterial community composition at phylum level.

**Figure 2:**
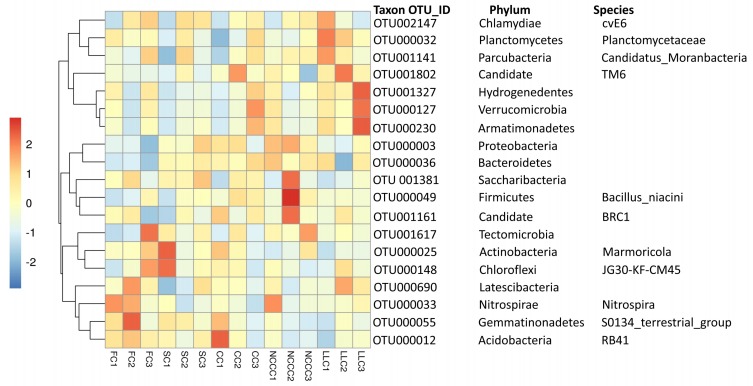
Heatmap analysis of abundant bacterial taxon under different planting system.

For all soil samples, 90.0–95.0% of the total reads were classified as top 10 bacterial classes and phyla. In all soils, the bacterial communities mainly consisted of members of the classes Alphaproteobacteria (13.0–16.45%), Gammaproteobacteria (12.09–19.97%), Gemmatimonadetes (6.17–10.67%), Planctomycetacia (1.69–13.34%), and Bacilli (4.70–10.39%), while the consistent phyla were dominated by Proteobacteria, Actinobacteria, Planctomycetes, Gemmatimonadetes, Firmicutes, Acidobacteria, Chloroflexi, and Bacteriodetes. The class of Proteobacteria was dominated by Alphaproteobacteria (16.45%), Gammaproteobacteria (19.97%), Deltaproteobacteria (7.36%), and Betaproteobacteria (7.60%), and significantly higher relative sequence abundance was observed in NCCC1, NCCC2, FC2, and CC1 soil, respectively (Figure 1A). Among phyla sequences, phylogenetic relative abundance analysis demonstrates that the NCCC2 treatment had the highest relative abundance of Proteobacteria (44.70%), followed by NCCC1 and SC3, while Actinobacteria (17.30%) was most abundant in SC1, followed by the CC1, FC3, NCCC3, and CC2 treatments (Figure 1B). The highest abundance (17.98%) of Planctomycetes was identified in LLC1, and the lowest abundance (3.9%) in CC1 treatment. The FC2 treatment had the highest relative abundance (13.90%) for Gemmatimonadetes, and LLC1 treatment had the lowest abundance (7.81%). Firmicutes (10.86%) and Acidobacteria (7.89%) phyla were significantly abundant under NCC2 and CC1 treatments, respectively. Chloroflexi (4.19%) and Bacteroidetes (3.57%) were shown to be significantly more enriched in SC1 and NCCC1, respectively, compared to FC treatment. Other minor bacterial communities, such as TM6 (candidate), BRC1 (candidate), RB41 (acidobacteria), Chlamydiales, and Tectomicrobia, were also observed in the cucumber rhizosphere soil under different planting systems (Figure 2).

**Figure 3:**
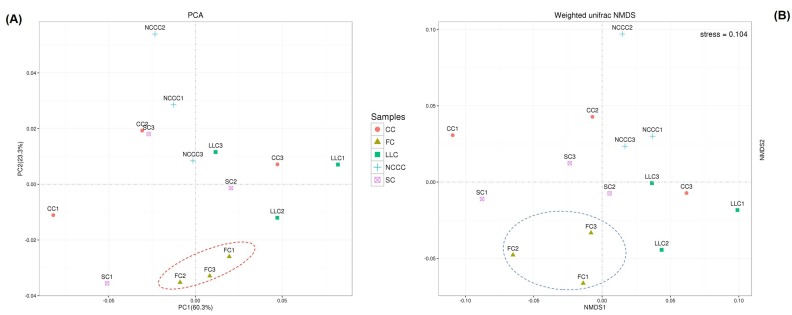
Sample sorting analysis revealed the microbial community ordination plots for *16S rRNA* bacteria: (**A**) scatter plots based on UniFrac phylogenetic distances with principal component analysis (PCA) ordination (PC1 vs PC2), sampling differences with non-metric multidimensional scaling of Unifrac weighted non-metric multidimensional scaling (NMDS) (**B**).

**Figure 4:**
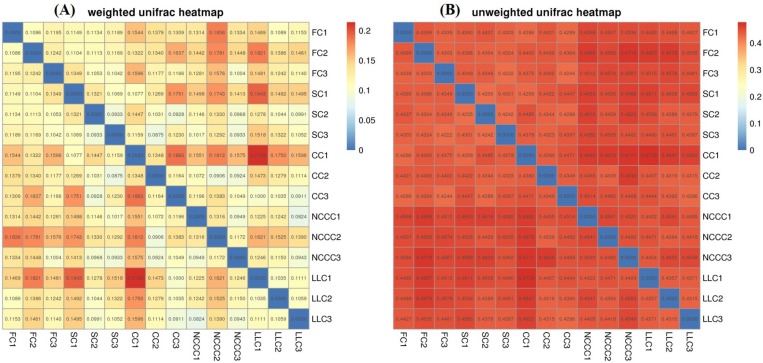
Heatmap based distance matrix of beta diversity analysis using weighted uniFrac (**a**) unweighted uniFrac (**b**).

We observed different phyla distributions among the samples, and differences in the composition of bacterial communities between all samples were visualized based on UniFrac unweighted pair group method with arithmetic mean (UPGMA) in combination with clustering analysis at both levels (Figure 1). The hierarchical clustering results revealed that the rhizosphere soil microbial community profiles relatively differed and bacterial communities could be significantly affected by different planting systems. For example, cluster analysis of the bacterial community structure of FC2, FC1, CC1, and SC1 treatments together resulted in a major cluster that was clearly differentiated from rest of the treatments. CC2 and SC3 treatments clustered within the same group, separate from the LLC treatment. Further general differences in the composition of bacterial communities (*16S rRNA* gene sequencing) among the different samples were also confirmed by principal component analysis (PCA) and non-metric multidimensional scaling (NMDS) ordination. The first and second PCA variation (60.3% for PC1 and 23.3% for PC2) accounted for a cumulative variance sum of 83.6% across all samples (Figure 3A). There were significant differences between fallow–cucumber and species-planted soil. The bacterial community of the FC treatment grouped well and exerted a distinct impact as compared to the communities of other treatments. NMDS based on Bray–Curtis distances among samples also revealed apparent differences among groups (Figure 3B). The bacterial communities from the planting system of cover crops clustered separately with communities from FC groups on the NMDS plot. A heatmap of beta diversity index was used to measure the dissimilarity coefficient between two samples. Weighted and unweighted UniFrac distances indicated the close proximity and some general differences of the bacterial communities in the three replicates (Figure 4a,b).

### 2.4. Comparative Assessment of Microbial Biomarkers

**Figure 5:**
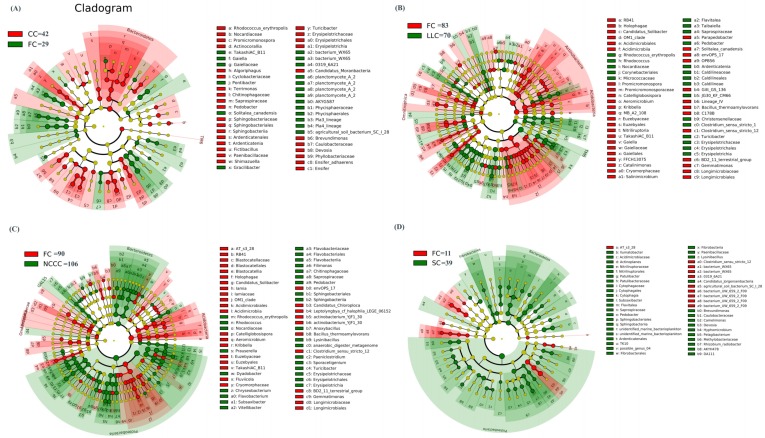
Cladogram plotted from LEfSe comparison analysis indicating the taxonomic representation of statistically and biologically consistent differences of identified biomarkers among different cropping systems. (**A**) Spinach–cucumber vs. fallow–cucumber (CC-FC); (**B**) fallow–cucumber vs. leafy lettuce–cucumber (FC–LLC); (**C**) fallow–cucumber vs. non-heading Chinese cabbage–cucumber (FC–NCCC) and (**D**) fallow–cucumber vs. spinach–cucumber (FC–SC). The colored shadows represent trends of the significantly differed taxa. The red or green shading depicts bacterial taxa that were significantly higher in each cropping system whereas species with no significant difference are uniformly colored to yellow.

We used the linear discriminant analysis effect size (LEfSe) method for quantitative analysis of biomarkers within different groups and to further elucidate the possible interactions of the identified bacterial affiliations in soil samples. Through the detection of significant differences (linear discriminate analysis (LDA) > 2; *p* < 0.05) in the abundance of different bacterial biomarkers within the groups, the cladogram revealed that 470 biomarkers were identified in all soil samples. Across all taxonomic levels of FC–CC, 42 biomarkers were associated with CC and 29 were associated with FC groups (Figure 5A). The higher abundance of Bacteroidetes species (LDA 3.53, *p* = 0.04), including Sphingobacteria and Sphingobacteriales families, were significantly enriched in CC groups (Figure 5A; Supplementary File S2). Actinobacteria were highly abundant biomarkers identified in the FC–LLC dataset, and species including *Corynebacteriales*, *Nocardiaceae*, *Nocardia*, and *Nocardia farcinica* (LDA 4.12, *p* = 0.04) were found to be significantly abundant in FC groups (Figure 5B; Supplementary File S2). The maximum number of discriminant clades significantly increased in NCCC (106) and SC (39) groups (Figure 5C; Supplementary File S2; Figure 5D; Supplementary File S2). Proteobacteria were the potential taxon indicators that primarily changed under these planting systems, with an LDA score of 4.51 and 4.01, respectively at *p* = 0.04 level. (Figure 5C; Supplementary File S2; Figure 5D; Supplementary File S2).

### 2.5. Soil Bacterial Diversity Responses to Different Planting Types

The bacterial α-diversity of the groups varied greatly among the five planting systems. The total number of OTUs (5119 OTUs) in the SC treatment was the largest among the communities (Table 4). Further, microbial differences in soil samples were revealed by comparing the richness and diversity indices. Richness indices in the SC treatment (Ace 7048; Chao 7016) were highest, and were significantly higher with respect to other treatments. According to OTU diversity estimated by Shannon’s index, the greatest bacterial diversity was in the SC (9.98) and NCCC (9.91) treatments, whereas the difference was not significant for the Simpson index under any treatments. In addition, we also noted that OTUs, richness, and Shannon estimator constantly decreased with the LLC treatment compared to other treatments (Table 4). These results indicate that some cropping systems are diversified in their role of inducing soil bacterial diversity in cucumber-planted soil.

### 2.6. Linking Bacterial Community to Soil Properties

**Figure 6:**
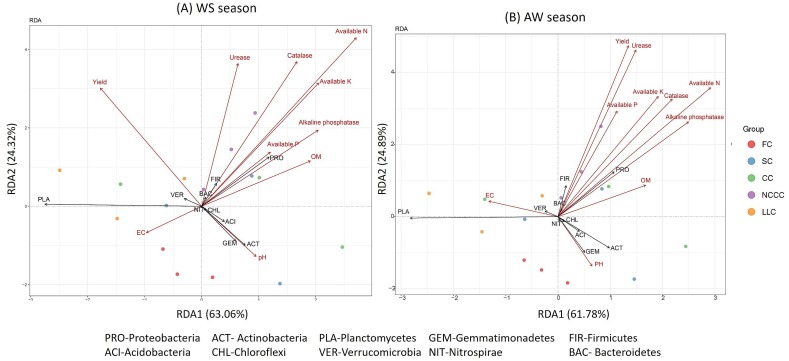
Redundancy analysis (RDA) of soil bacterial community structure associated with soil properties. (**A**), RDA derived from WS season-2017 samples; and (**B**), RDA derived from AW season-2017 samples.

Redundancy analysis indicated relationships between soil environmental factors (soil properties) and bacterial community composition, and results show that the relative abundance (>0.5% average richness) of bacteria was affected by both growing season and soil properties (Figure 6). For the WS season, first and second axes show that 63.06% and 24.32% of changes in the bacterial community were influenced by soil properties (Figure 6A). The significant abundance of Proteobacteria and Firmicutes under the NCCC and SC plots was associated with soil biological indicators (catalase, urease, alkaline phosphatase) and nutrient content (available P). WS yield was significantly induced by Verrucomicrobia under the LLC treatment. The slight change in pH level was significantly associated with the change of Acidobacteria, Gemmatinonadetes, and Actinobacteria, while soil EC had no significant effect on the distribution of bacterial communities (Figure 6A).

For the AW season, the first two axes of the redundancy analysis (RDA) explain 61.78% and 24.89% of the total variance in the major bacterial groups. A majority of soil properties had a significant impact on Proteobacteria and Firmicutes abundance. Soil pH affected the relative abundance of Acidobacteria and Gemmatinonadetes. Soil EC had a significant effect on Verrucomicrobia abundance. Planctomycetes abundance was not affected by any environmental factors.

### 2.7. Predictive Metagenomics Profiling

**Figure 7:**
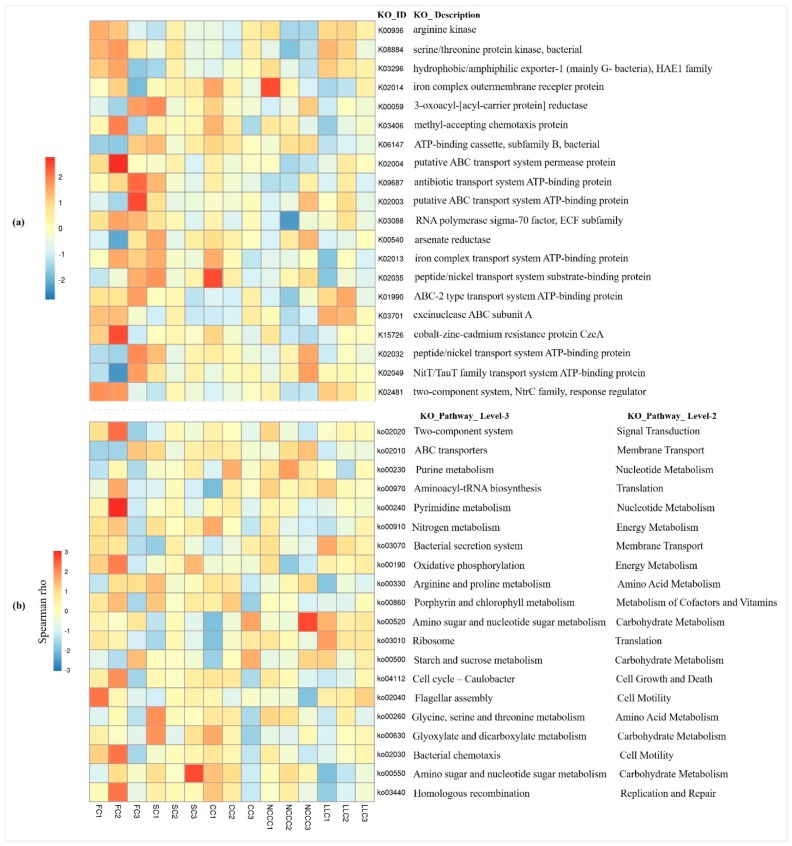
Heatmap based most abundant Kyoto Encyclopedia of Genes and Genomes (KEGG) ortholog (KO) groups in microbiome samples (**a**) Imputed metagenomes reveal the relative abundance of only top 20 KEGG metabolic pathways across all the soil samples (**b**). KEGG metabolic pathways correlating positively or negatively within abundant genes at *p*-value < 0.05.

We further used the novel Tax4Fun tool, which explains the predictive functional profiling of microbial communities using *16S rRNA* marker gene sequences. Results indicated the predicted Kyoto Encyclopedia of Genes and Genomes (KEGG) functional categories from the gene contents and abundances among microbial communities. Approximately 45.00–55.46% of the total sequences were assigned to specific KEGG orthologs, indicating a high number of sequences associated with arginine kinase (K00936), iron complex outer membrane receptor protein (K02014), methyl-accepting chemotaxis protein (K03406), iron complex transport system ATP-binding protein (K02013), and arsenate reductase (K00540) across all soil samples (Figure 7a). Also, predictive KEGG pathways revealed assigned sequences mainly associated with metabolism, genetic information processing, environmental information processing, and cellular processes at level 1 (Figure 7b).

According to KEGG pathways at levels 3 and 2, functional profiles by the LLC group were related to amino sugar and nucleotide sugar metabolism (carbohydrate metabolism). The NCCC group mainly increased the function of purine metabolism (nucleotide metabolism), while the CC group mainly increased the function of nitrogen metabolism (energy metabolism). The metabolic functions related to arginine and proline, glycine, serine, and threonine metabolism (amino acid metabolism) were significantly higher in the SC group (Figure 7b). Two-component system (signal transduction), oxidative phosphorylation (energy metabolism), and aminoacyl-tRNA biosynthesis (translation) were the putative KEGG pathways primarily defined by the FC group. These metabolic profile indicate differential regulation of the soil bacterial community functional profiles by the different planting systems.

## 3. Discussion

Having sustainable PGVC in intensified areas is very important and provides important ecosystem services at the local, regional, and even global scale beyond increased food production. However, the current soil management practices and conventional cropping systems for this type of cultivation are not eco-physiologically sound. Therefore, innovative practices such as diversified cropping have emerged to reduce the impact of agriculture on climatic and environmental changes. We have chosen different cropping models during fallow periods of cucumber double-cropping to evaluate ecological and metabolic effects of leafy vegetables.

### 3.1. Effects of Different Cropping Systems on Soil Quality and Cucumber Yield

We found that soil pH was not changed significantly under any cropping system during the two growing seasons (Table 1 and Table 2), and this was likely caused by a short-term cover cropping effect and the complexity of the greenhouse environment. However, some treatments provoked the efficient cropping system to improve the soil quality components. Thus, introducing spinach–cucumber (SC) and non-heading Chinese cabbage–cucumber (NCCC) to the cropping system enhanced the amount of organic matter compared to the fallow–cucumber (FC) system, suggesting that the active carbon content in residual treatment can be considered as an available carbon fraction for microbes that are positively affected by the presence of these cropping treatments. In cropping systems, the addition of soil organic matter is considered a good soil quality indicator, and plant residue quality and quantity can greatly influence the soil organic carbon [10]. In fact, the soil organic matter (SOM) content between the start of the experiment (2016) and the date of sampling (2017) showed an increase of 7.35 and 9.13 g/kg under SC and NCCC for WS and AW seasons, respectively. The incorporation of these planting systems may have led to a sufficient return of crop residues to allow this increasing trend in soil organic matter in the past 7 years of continuous replanted soil, which earlier showed low SOM. The results were in line with previous findings, where a pronounced effect on organic matter content was attained after pea–vetch cropping rotation [11]. A similar impact on rotational scale was examined in [26], showing that the shift from cereal-fallow to wheat–faba bean rotation provided rich residue for higher carbon stock as it shifted from conventional tillage to no tillage.

Soil nutrient contents and biological activities are the core quality indicators for the below-ground microbial community and diversity that are greatly affected by the cropping system. Soil available nutrient contents and enzyme activities were significantly induced by our cropping system for both growing seasons. The highest available P in the SC planting system was likely due to the highest alkaline phosphatase activity in the WS season, suggesting an appropriate nutrient cycling capacity and OM turnover [38]. The available nutrients pools (NPK) in our studied soil under the next AW season were greatly influenced by the NCCC planting system, suggesting that further nutrient cycling and associated enzyme activity (urease, catalase, and alkaline phosphatase) were more conducive in this cropping system [39]. The capacity to reduce nitrate leaching by capturing N from preceding crops as well as available N in the rhizosphere might be expected from certain non-leguminous cover crops [12]. In line with this concept, leafy crops based on the SC and NCCC planting systems corroborate the direct effects of the quantity and quality of crop residue that could be supposed as a premise of organic matter input, soil biological function in continuous replanted soil. The results were in line with previous short-term findings that significant changes in soil properties could be expected in various agronomic conditions such as cropping systems, soil health, and organic input [1,2,3].

The seasonal cucumber yield was evidently promoted by greater biomass under the SC cropping system in the WS season, followed by a large impact of the NCCC planting system in the AW season. The results indicate that higher yield response was likely caused by greater nutrient deposition and soil biological activation, and these cropping models were beneficial for increased cucumber yield and relief of continuous cropping–based soil sickness. A similar yield-promoting effect was also observed with tomato–celery–cucumber [40], soybean–pigeon pea rotation [34], wheat–cucumber companion cropping [3], and garlic–cucumber [41] systems. The lowest cucumber yield was found with leaf lettuce–cucumber (LLC) during the latter growth period (AW season), and this effect was likely due to the competitive interaction among neighboring plants for resources and nutrients [42]. In addition, Chinese cabbage produces glucosinolates, which are secondary metabolites. In Brassicaceae cover crops, glucosinolates and their hydrolysis products can inhibit soil-borne pathogens, including Fusarium oxysporum of cucumber, and can affect cucumber yield [36].

### 3.2. Effects of Different Cropping Systems on Soil Bacterial Diversity

Soil microbial community (SMC) composition and diversity are generally considered as important soil health indicators. Plant species, root exudates, and soil types are key determining factors that shape and drive the succession of the soil microbial community structure [39,43,44]. Previous studies reported that an extended monoculture period caused significant reduction in microbial diversity in cucumber-planted soil [35]. Our greenhouse field was previously established with long-term continuous cucumber cultivation, and negative soil-plant feedback of CCO is known for SMC response. In the current study, discernible separation in the rhizosphere microbial community structure among the one-year newly planted soil and 7-year monocultured soil were investigated for the cucumber double-cropping system. We suggest that most of the richness and diversity indices were significantly changed among the rhizospheres of various planting systems, indicating the driving force of diverse planting systems in the SMC structure. The SC and NCCC cropping system were the most conducive to the formation of soil microbial colony diversity among all the cropping systems analyzed, especially in terms of OTUs, species richness (ace, Chao), and bacterial diversity measured by the Shannon diversity index (Table 4), in agreement with [20], which reported that the highest level of bacterial diversity forcefully changed under different summer cover cropping systems. Greater bacterial diversity with cover cropping systems (cucumber–Chinese cabbage and cucumber–celery) in the cucumber rhizosphere was identified in [45]. In [46] it was shown that higher plant diversity could alter the specific microbial population, and rape–cucumber and mustard–cucumber companion systems stimulated the bacterial richness, evenness, and diversity of monocropped cucumber-planted soil.

The plant density of the SC and NCCC cropping systems engaged higher bacterial diversity, which may have mainly been due to the release of distinct profiles of root exudates. Consistent with previous studies [47,48], it is acknowledged that root exudates from particular plant species /genotypes initiate and modulate the interactions between roots and soil microbes that degrade organic substrate and provide a carbon source to soil microorganisms, stimulating beneficial symbiosis. Such below-ground interactions thus change the soil properties, which may also determine the microbial diversity around the rhizosphere [49]. These data suggest a possible preference of leafy vegetable crops to assemble different SMCs, and a temporal shift in the abundance of microbial diversity was due to both the different cover crop communities and differences in the quality of plant inputs. Additionally, it is noted that the fallow–cucumber (FC) cropping system was characterized by relatively more bacterial diversity as compared to the LLC cropping system, which also confirms that reducing agricultural cropping can also increase site-specific soil microbial diversity, as reported by previous studies on single-cropping [50,51].

### 3.3. Changes in Community Composition and Functional Profiling of Active Microbiome

At the phylum level of SMCs, the dominant bacteria (i.e., relative abundance) in the various planting systems were mainly distributed in Proteobacteria, Actinobacteria, Planctomycetes, Gemmatimonadetes, Firmicutes, Acidobacteria, Chloroflexi, and Bacteroidetes. In a wider context, PCA and NMDS analysis reveals that a cover cropping system with leafy vegetables could be more effective in shaping the SMC structure than the FC system. Further, discriminant biomarkers in the rhizosphere of FC vs. CC, FC vs. LLC, FC vs. NCCC, and FC vs. SC were significantly retrieved by LEfSe analysis (Figure 5). Significantly more abundant biomarker species were found in planted soil of the CC (42 clades), NCCC (106 clades), and SC (39 clades) cropping systems compared to the FC planting system. The differences among the identified genetic indicators in the different cropping systems suggest that Proteobacteria, Actinobacteria, Bacteroidetes, and Acidobacteria were the potentially functional and active soil microbiomes in our planting system. This parallels the findings of [52], which identified the same bacterial species under different plant species, indicating that the functional capabilities of these microbiomes can largely affect the structural and functional diversity of microbial community composition.

In addition, soil-derived determinants could be important indicators that correlate with soil bacterial communities, as shown by RDA analysis (Figure 6A,B). Our results agree with previous studies [36,53], suggesting that significant changes in soil nutrient and biological functions are likely associated with changes in bacterial community composition and diversity. In this study, we found that soil pH was the main factor, and a slight change in pH significantly correlated with several core phyla (Gemmatimonadetes, Acidobacteria, and Actinobacteria). Previous reports suggested that many bacterial activities, such as ammonia oxidization and phosphate solubilization, are pH-dependent [54,55]. Acidobacteria contain many taxa that have physiological functions that are pH-dependent [36,53], which might explain the large impact of pH on those bacteria.

### 3.4. Effects of Compositional Shift on Predictive Metabolic Functions

The quantitative analysis of abundant genes and biological pathways provides key insights into the metabolic capabilities of microbial communities [37]. Using metagenomic statistical analysis tools, we predicted the possible effects of these community composition shifts on potential functions. The significant functional differences under the different cropping systems are presented in a heatmap (Figure 7). The higher the abundance of genes, the more complementary metabolites and putative pathways were found in all cropping systems, suggesting that the affiliated metabolic exchange interaction was likely driven by different planting species.

Among the classified metabolic functions, the resulting pathway of metabolism was the most dominant category, including several metabolic pathways, including carbohydrate metabolism, energy metabolism, amino acid metabolism, and others. Our findings revealed that the cucumber rhizosphere was preferentially colonized by Proteobacteria and Actinobacteria, suggesting that the corresponding phyla formed a “core rhizosphere microbiome” that maintained various normal functions [56]. Previous reports indicated that the heightened abundance of Proteobacteria and Actinobacteria is especially conducive to carbon metabolism, membrane transport system including ABC transport, and the stress response regulatory system [56,57]. Several members of Proteobacteria and Actinobacteria in our study were thought to be fast decomposers for labile C compounds and plant polymers [58]. Within these phyla, the genera *Bradyrhizobium, Pseudomonas, Rhodopseudomonas*, and *Mycobacterium* were identified in the cucumber rhizosphere, and these genera harbored diverse functional genes for nutrient transformation [55]. The affiliated strains of *Arthrobacter* (Actinobacteria) are suggested to produce an antimicrobial effect within species by producing volatile organic compounds against pathogenic bacteria [59]. In particular, potential nitrogen metabolism (KEGG level 3) in our study indicate the functional capacity of the CC3 planting system with respect to N metabolism, considering that the coriander planting species is supposed to regulate the function of N acquisition by N-cycling microbes, and their effect might be associated with the specific chemical composition of the organic residues [60]. This is consistent with members of Proteobacteria, possibly leading to more active microbially mediated N transformation in the rhizosphere [61]. *Mycobacterium* genera are involved in autotrophic carbon fixation and antibiotic resistance [62]. Interestingly, the Tax4Fun KEGG pathway predicted an elevated activity of arsenic metabolism and detoxification by different planting systems. Arsenic (As) is a naturally occurring toxic metalloid, and previous reports indicated that As accumulation in plants resulted in alteration of the physiochemical and biological properties and, consequently, loss of crop yield [63]. Arsenate detoxification is mainly caused by arsenate reductase enzymes, which have been suggested to reduce the As biosynthesis pathway [64]. Several plant species have been reported to monitor the degree of arsenic, and their intrinsic ability has already been determined for As phytoremediation [65]. However, it is not clear which microorganisms play key roles in the transformation of As. We suggest that the planting with NCCC3 and SC1 species increases the function of arsenate reductase, indicating that spinach and non-heading Chinese cabbage root exudates are capable of converting inorganic arsenic into volatile organic arsenic, reducing the adverse effect of As accumulation in main cropping biomass. This observation was consistent with a previous report revealing that the plant roots are capable of rapidly taking up arsenite from an external medium [66]. Conversely, enzyme activity was significantly reduced under the FC2 system. As-contaminated groundwater, continuous cropping based anthropogenic inputs, and As-containing agrochemicals are thought to lead to downstream deposition of As in our intensive PGVC production [15,16].

Metabolic information regarding the effects of different cover planting systems on SMC functional profiles is currently very scarce. Our predicted metabolic characteristics could be an important ecological signal to provide direct evidence of a community’s functional capabilities. To date, no significant reports have delineated a strong co-occurrence pattern of environment-specific metabolic functions in the context of the current analysis. Further, quantitative modeling of complex metagenomic community functions should be framed for more winter catch leafy vegetable species under intensive cropping systems.

## 4. Materials and Methods

### 4.1. Field Description and Experimental Site

Field experiments were conducted between 15 November 2016 and 20 November 2017 at Yangling Agricultural Hi-tech Industries Demonstration Zone, a commercially intensified region of PGVC located in Northwest China in a semi-humid temperate climate. The study site (34°17′N, 108°04 E) was the experimental station of the College of Horticulture, Northwest A&F University. The trials were conducted under a typical 8-year-old commercial plastic tunnel (double-cropping planting system) covered with plastic film (ground surface area 8 m × 60 m) without supplementary lighting or heating and oriented in a north-south direction. The mean air and soil temperatures were 23.3 and 19.4 °C in the spring season and 17.3 and 16.1 °C in the autumn season, respectively, under the greenhouse structure.

The surface soil in the plastic greenhouse (0–30 cm) was classified as Orthic Anthrosol (FAO soil taxonomy) with a sandy loam texture. Prior to the experimental periods, the commercial PGVC site had been cultivated for long-term continuous cucumber monoculture with a double-cropping system for 7 years [67]. The field soil was recognized as continuous replanted and had a pH value of 7.76, containing 15.59 g/kg organic matter, 1.43 g/kg total N, 0.93 g/kg total P, 7.15 g/kg total K, 53.65 mg/kg available N, 59.41 mg/kg available P, and 305.91 mg/kg available K.

### 4.2. Experimental Design, Crop Establishment and Management

In both years, field experiments were laid out in a randomized block design with 3 replications under a plastic tunnel greenhouse. Three beds were assigned for each plot and the average size of each replicate plot was 12.96 m^2^ (3.6 m wide × 3.6 m long). The selected leafy vegetables as winter catch cover crops were introduced during the winter fallow period of cucumber in November and harvest in February, followed by immediate planting of spring seasonal cucumber. The leafy crops cultivated in the cucumber planting system were spinach (*Spinacia oleracea*), coriander (*Coriandrum sativum* L.), non-heading Chinese cabbage (*Brassica rapa* ssp. *pak choi*), and leafy lettuce (*Lactuca sativa* L.). The study consisted of 5 treatments: fallow–cucumber (FC), spinach–cucumber (SC), coriander–cucumber (CC), non-heading Chinese cabbage–cucumber (NCCC), and leafy lettuce–cucumber (LLC). Impermeable plastic film was embedded into the soil between studied plots to a depth of 50 cm and extending above the ground by 5 cm. The plastic sheets were used to avoid the mutual influence among treatments by preventing lateral and transverse migration of nutrients and water between plots.

During the off-season of previous cucumber cultivation (fallow period: 20 November to 15 February 2016), 4 over-wintering leafy vegetables were incorporated in each plot, except the FC plot, which was left fallow until the next growth period of cucumber cropping. After a short-term overwinter season, all the leafy vegetables were harvested at their leaf growth stage, above- and below-ground biomass was chopped and incorporated into the soil, and plots were manually tilled and prepared for the next cucumber cultivation. Uniform cucumber seedlings (*Cucumis sativus* L. cv. Jinglu No. 3) with 2 leaves were transplanted (0.6 m spacing between rows, 0.30 m between plants) from mid-February to June as the first growing season (winter–spring cultivation, WS) and from August to October as the second growing season (autumn–winter cultivation, AW) in 2017. During WS and AW cultivation in the same year, each plot received a compound NPK fertilizer (16:16:8) at a rate of 300 kg ha^−1^. Half of the inorganic fertilizer was applied basal, and the rest was dissolved and dressed in the furrow irrigation water. No chemical fertilizers were applied during leafy vegetable plantation. The same fertilization and management were performed for all study plots during the entire experimental period.

### 4.3. Soil Sampling and Analysis

Rhizosphere soil samples were taken after WS and AW cultivation to check the responses of different cropping systems. Molecular characterization of soil bacterial communities, diversity structure, and metabolic profile was carried out after the final harvest of cucumber in November 2017. Soil sampling was obtained as described by our previous research group [67]. In brief, soil that was tightly adhered to roots was considered rhizosphere soil, and cucumber rhizosphere soil samples were collected from 3 random points within each replicate plot and mixed together to make the composite samples, and 3 biological replicates were obtained for each treatment. A total of 15 soil samples were obtained each time and immediately transported to the laboratory on ice. The soil samples were sieved (<2 mm), thoroughly homogenized, and divided into 2 subsamples: one was stored at −40 °C for measurement of soil properties, and the other was stored at −80 °C for DNA extraction and subsequent microbial analysis.

Soil physiochemical analysis methods were used according to [68]. Soil pH and EC were determined in a soil/water suspension (1:2.5 *w*/*v*) using a glass electrode and a conductivity meter, respectively. Soil organic carbon (SOC) was measured with the dichromate oxidization method followed by FeSO_4_ titration. Total N (TN) was determined using the Kjeldahl method. Measurements of soil AP and AK were determined using Olsen’s and flame photometric techniques, respectively. The activity of 4 soil enzymes (invertase, urease, catalase, and alkaline phosphatase) was measured following the procedures as described in [67,69]. The activity of soil catalase (EC 1.11.1.6) was measured with H_2_O_2_ (0.3%) as a substrate, and filtrate suspension was titrated with 0.1 mol L^−1^ KMnO_4_ mL g^−1^ soil 20 min^−1^. Soil urease (EC 3.5.1.5) and invertase (EC 3.2.1.26) activities were determined by using 10% urea and 8% glucose solution, respectively, as the substrate, and values were expressed as products per gram of dry weight soil mass per incubation time (24 h). Soil alkaline phosphatase (EC 3.1.3.1) activity was determined by the addition of 10 mL disodium phenyl phosphate solution as a substrate and phenol as a product after incubation at 37 °C for 24 h.

### 4.4. DNA Extraction, Polymerase Chain Reaction (PCR) Amplification, and Metagenomic Sequencing

High-throughput sequencing analysis of the *16S rRNA* gene was performed to determine soil bacterial diversity and community. Total genomic DNA was extracted from 0.5 g soil using the E.Z.N.A. soil DNA Kit (Omega Biotek, Norcross, GA, USA) according to the manufacturer’s protocol. Three replicate DNA extractions were performed for each soil sample. The concentration and quality (A260/A280 ratio) of the DNA samples were determined using a NanoDrop 2000 spectrophotometer (Thermo Scientific, Waltham, MA, USA).

Polymerase chain reaction (PCR) amplification was carried out as described in [70]. Briefly, the hypervariable region V3–V4 of the *16S rRNA* genes was amplified using universal primers 341F(5′-CCTACGGGNGGCWGCAG-3′) and 806R (5′- GGACTACHVGGGTATCTAAT-3′, where the barcode is an eight-base sequence unique to each sample. The PCR reaction was carried out in triplicate with 50 μL mixture containing 5 μL of 10 × KOD buffer, 5 μL of 2.5 mM dNTPs, 1.5 μL of each primer (5 μM), 1 μL of KOD polymerase, and 100 ng of template DNA. The PCR conditions were 95 °C for 2 min, followed by 27 cycles at 98 °C for 10 s, 62 °C for 30 s, and 68 °C for 30 s, and a final extension step at 68 °C for 10 min.

Amplicons were cleaned up using AMPure XP beads (Beckman Coulter Inc., Brea, CA, USA) and quantified using Qubit 3.0 with Qubit dsDNA HS Assay Kit (Thermo Fisher Scientific, Waltham, MA, USA) according to the manufacturer’s instructions. The triplicate amplification products were pooled and quantified using NanoDrop (Thermo Scientific, USA. Subsequently, prepared libraries were sequenced on an Illumina HiSeq 2500 PE 250 platform at Gene Denovo Biotechnology Co. Ltd. (Guangzhou, China). The raw sequences data generated in this study were deposited in NCBI under Bioproject PRJNA with the accession number of 543220.

### 4.5. Sequence Data Analysis, Bioinformatics

Paired-end reads of the *16S rRNA* gene were assembled using Flash (v1.2.11) (http://ccb.jhu.edu/software/FLASH/) to obtain raw tags. The raw tags were filtered using QIIME2 pipeline under specific filtering conditions [71]. Clean tags were searched against the Gold database (http://drive5.com/uchime/uchime_download.html), and chimeric sequences classified as non-amplified regions or low-quality fragments were detected and eliminated using the UCHIME algorithm (http://www.drive5.com/usearch/manual/uchime algo.html). After chimeric checking and removal, filtered sequences were clustered into operational taxonomic units (OTUs) at 97% sequence similarity using the UPARSE pipeline [72]. The taxonomic classification of each representative *16S rRNA* gene sequence at different taxonomic levels (from phylum to genus) was analyzed by the RDP Classifier algorithm (http://rdp.cme.msu.edu/) based on the Silva (SSU123) database (https://www.arb-silva.de/).

### 4.6. Predictive Functional Profiling of Microbial Communities Using *16S rRNA* Gene

To provide a good functional approximation of reference sequences obtained through *16S rRNA* gene, Tax4Fun, an open-source R package that predicts the functional capabilities of microbial communities based on 16S datasets [37], was used. For the Silva–Tax4Fun approach, the Silva-labeled OTU table was used to investigate predictive functional attributes of microbial communities. Briefly, Tax4Fun converts the Silva-labeled OTUs into prokaryotic KEGG organisms, and then normalizes these predictions by the *16S rRNA* copy number (obtained from the National Center for Biotechnology Information (NCBI) genome annotations). The predictive functions of the microbial communities were determined by linearly combining the normalized taxonomic abundances with the precomputed association matrix of KEGG Ortholog reference profiles to Silva-defined microorganisms constructed by Tax4Fun (v1.0). The estimated metagenomic functional profile can be further utilized to infer the metabolic profile followed by this R method.

### 4.7. Statistical Analysis

The observed data were analyzed by using one-way analysis of variance (ANOVA) according to the model randomized block design with a 95% confidence interval. To see a real difference in the variables of crop yield, soil physicochemical properties, and α-diversity due to the treatment, we performed least significant difference test at the 5% level through the SPSS Statistics package for Windows (SPSS, version 18.0, Chicago, IL, USA). UPGMA clustering analysis was used to indicate the similarity distance between different community structures, and the results of clustering were integrated with the relative abundance of species at all levels. Statistical analysis of OTU richness via Good’s coverage, Chao1, and Shannon’s index was performed with Mothur (version 1.22.2). Multivariate statistical techniques including PCA, principal coordinate analysis (PCoA), and NMDS were employed for sampling differences. Biomarker features in each group were screened by Metastats and LEfSe software. For LEfSe analysis, a Kruskal–Wallis rank sum test and Wilcoxon rank sum test were used for multiple sample comparison and for group comparison of two samples, respectively. The LEfSe algorithm explains the quantification of most biologically informative organisms, genes that characterize the differences among planting treatment samples. Redundancy analysis (RDA) was performed to determine which environmental factors were related to the soil microbial community composition.

## 5. Conclusions

The promotion of ecosystem health in disturbed plant–soil systems relies upon our ability to identify the key ecosystem functions that must be restored. Therefore, site-appropriate crop modelling with leafy vegetables as cover crops incorporated in the conventional cropping system can assist in creating sustainable soil quality feedback. Overall, we found that increasing the diversity of cropping systems during the cucumber–fallow period was more favorable in degraded soil, and the cropping system affected the soil properties and microbiome structure. The spinach cropping (SC) system favored the accumulation of OM, available P, and soil biological functions for cucumber yield during the WS season. The non-heading Chinese cabbage (NCCC) cropping system manipulated OM decomposition, nutrient cycling, and soil biological processes for greater cucumber yield during the AW season. The soil quality feedback effect consequently altered the microbiome structure and induced soil bacterial diversity. Many taxa with significant metabolic potential predominated in our cropping system, suggesting that core soil bacteria are important in physiological regulation as well as in abating the environmental stress response in plants. This study fills a gap of knowledge on metabolic capabilities and predictive functional annotations of specific microbial groups in intensive production areas. Moreover, considering the greater capabilities of leafy cover crops, this study provides guidance for selecting the best cropping model during fallow periods of cash crops. This concept could be extended by incorporating more representative species into the cropping system to gain a more general understanding of their ecological roles in plant microbial-driven below-ground processes.

## Figures and Tables

**Table 1:** Effects of different winter catch planting system on soil physicochemical and biological characteristics of winter–spring (WS) season-2017.

Physicochemical and Biological Factors	Treatments				
	FC	SC	CC	NCCC.	LLC
Soil pH	7.74 ± 0.12 a	7.76 ± 0.05 a	7.76 ± 0.03 a	7.71 ± 0.05 a	7.73 ± 0.03 a
Electrical conductivity (EC) (µs·cm^−1^)	627.7 ± 5.00 bc	649.9 ± 5.90 a	621.6 ± 3.51 c	632.40 ± 4.79 bc	641.40 ± 1.79 ab
Organic matter (OM) (g·kg^−1^)	18.63 ± 1.28 c	22.94 ± 1.09 a	22.71 ± 1.23 a	21.55 ± 1.29 ab	19.45 ± 1.59 bc
Available N (mg·kg^−1^)	122.8 ± 3.03 c	144.4 ± 1.18 a	137.44 ± 1.59 ab	146.39 ± 1.77 a	132.69 ± 3.83 b
Available P (mg·kg^−1^)	64.39 ± 2.00 c	78.29 ± 0.75 a	73.25 ± 2.25 ab	69.52 ± 0.19 bc	68.12 ± 1.39 bc
Available K (mg·kg^−1^)	345.4 ± 1.73 d	354.78 ± 2.25 bc	363.06 ± 0.89 a	359.89 ± 1.85 ab	351.49 ± 3.82 cd
Soil invertase (mg·g^−1^ soil d^−1^)	43.50 ± 1.36 b	45.38 ± 1.07 b	63.86 ± 1.39 a	59.34 ± 3.67 a	46.64 ± 1.17 b
Urease (mg·g^−1^ soil h^−1^)	3.61 ± 0.17 d	5.54 ± 0.11 a	4.46 ± 0.26 c	5.46 ± 0.65 ab	4.96 ± 0.41 bc
Catalase (mg·g^−1^ 20 min^−1^)	6.29 ± 0.22 c	12.04 ± 0.85 a	9.82 ± 0.27 b	11.85 ± 0.67 a	9.15 ± 0.24 b
Alkaline phosphatase (mg g^−1^ soil h^−1^)	21.43 ± 0.60 d	34.66 ± 1.09 a	29.65 ± 1.39 b	27.87 ± 0.38 b	25.21 ± 1.10 c

Treatment values (mean ± standard error; *n* = 3) within a row followed by different letters are significantly differences at *p* ≤ 0.05 levels according to the least significant difference (LSD) means comparisons test. Treatments: FC (fallow- cucumber); SC (spinach–cucumber); CC (coriander–cucumber); NCCC (non-heading Chinese cabbage–cucumber); LLC (leafy lettuce–cucumber).

**Table 2:** Effects of different planting system on soil physicochemical and biological characteristics of autumn–winter (AW) season-2017.

Physicochemical and Biological Factors	Treatments				
	FC	SC	CC	NCCC	LLC
Soil pH	7.73 ± 0.12 a	7.71 ± 0.04 a	7.73 ± 0.03 a	7.70 ± 0.02 a	7.73 ± 0.04 a
EC (µs·cm^−1^)	631.04 ± 6.81 bc	651.04 ± 5.46 a	627.82 ± 5.38 c	638.83 ± 3.7abc	645.51 ± 2.25 ab
OM (g·kg^−1^)	21.18 ± 1.58 ab	23.27 ± 0.99 ab	23.20 ± 0.54 ab	24.72 ± 1.00 a	20.09 ± 0.42 b
Available N (mg·kg^−1^)	117.28 ± 4.74 b	136.2 ± 5.43 a	138.9 ± 1.48 a	142.16 ± 1.67 a	123.89 ± 0.37 b
Available P (mg·kg^−1^)	57.72 ± 1.32 b	65.81 ± 2.60 ab	69.94 ± 2.00 a	72.39 ± 0.91 a	61.53 ± 5.03 b
Available K (mg·kg^−1^)	350.08 ± 1.76 cd	358.06 ± 0.91bc	365.84 ± 1.79 ab	368.58 ± 2.01 a	347.69 ± 5.40 d
Soil invertase (mg·g^−1^ soil d^−1^)	40.00 ± 0.56 b	41.86 ± 2.54 b	57.13 ± 2.51a	54.86 ± 2.08 a	38.99 ± 1.44 b
Urease (mg·g^−1^ soil h^−1^)	3.74 ± 0.25 c	5.88 ± 0.42 b	6.13 ± 0.43 ab	6.90 ± 0.16 a	4.760.19 c
Catalase (mg·g^−1^ 20 min^−1^)	5.72 ± 0.12 c	7.72 ± 1.23 bc	8.50 ± 0.84 ab	10.90 ± 0.86 a	7.61 ± 0.72 bc
Alkaline phosphatase (mg· g^−1^ soil h^−1^)	19.57 ± 0.57 c	21.87 ± 0.36 bc	24.28 ± 1.93 ab	25.68 ± 1.43 a	22.54 ± 0.70 ab

Treatment values (mean ± standard error; *n* = 3) within a row followed by different letters are significantly differences at *p* ≤ 0.05 levels according to LSD means comparisons test. Treatments: FC (fallow–cucumber); SC (spinach–cucumber); CC (coriander–cucumber); NCCC (non-heading Chinese cabbage–cucumber); LLC (leafy lettuce–cucumber).

**Table 3:** Effect of different planting system on seasonal cucumber yield (kg/plot).

Treatments	WS Season-2017	AW Season-2017
FC	46.21 ± 2.08 b	4.24 ± 0.46 bc
SC	55.23 ± 3.18 a	4.45 ± 0.59 bc
CC	50.82 ± 3.36 ab	5.36 ± 0.17 ab
NCCC	51.09 ± 2.54 ab	6.16 ± 0.06 a
LLC	48.65 ± 2.44 ab	3.72 ± 0.23 c

Treatment values (mean ± standard error; *n* = 3) within a row followed by different letters are significantly differences at *p* ≤ 0.05 levels according to LSD means comparisons test. FC: (fallow–cucumber); SC: (spinach–cucumber); CC (coriander–cucumber); NCCC (non-heading Chinese cabbage–cucumber) and LLC (leafy lettuce–cucumber).

**Table 4:** Characterization of soil bacteria richness and diversity indices in different cropping treatments under plastic greenhouse vegetable cropping (PGVC) conditions.

Sample ID	OTUs	Ace	Chao 1	Shannon	Simpson	Coverage %
FC1	4644	6134	6229	9.74	0.99	0.97
FC2	4726	6423	6319	9.48	0.98	0.96
FC3	4593	6027	5993	9.88	0.99	0.97
Average	4654.33 c	6195 c	6181 c	9.70 c	0.99 a	0.97 a
SC1	5407	7586	7452	10.07	0.99	0.96
SC2	4993	6785	6809	9.93	0.99	0.96
SC3	4958	6771	6788	9.92	0.99	0.96
Average	5119.33 a	7048 a	7016 a	9.98 a	0.99 a	0.96 a
CC1	4853	6860	6856	9.85	0.99	0.96
CC2	4982	6798	6768	9.68	0.99	0.97
CC3	4574	6081	6118	9.79	0.99	0.97
Average	4803 b	6580 b	6581 b	9.77 b	0.99 a	0.97 a
NCCC1	5021	6989	6860	9.99	0.99	0.96
NCCC2	4955	6865	6918	9.80	0.99	0.96
NCCC3	4667	6221	6146	9.95	0.99	0.96
Average	4821 b	6691 b	6441 b	9.91 a	0.99 a	0.96 a
LLC1	4270	5517	5597	9.76	0.99	0.97
LLC2	4603	6202	6188	9.89	0.99	0.97
LLC3	4554	6028	5955	9.81	0.99	0.97
Average	4475.66 d	5915 c	5913 d	9.82 b	0.99	0.97 a

Treatment values (mean ± standard error; *n* = 3) within a row followed by different letters are significantly differences at *p* ≤ 0.05 levels according to LSD means comparisons test. Treatments: FC (fallow–cucumber); SC (spinach–cucumber); CC (coriander–cucumber); NCCC (non-heading Chinese cabbage–cucumber); LLC (leafy lettuce–cucumber).

## Supplementary Materials

Supplementary materials can be found at https://www.mdpi.com/1422-0067/20/11/2619/s1.
