# Supplementary material for: Hiseq Base Molecular Characterization of Soil Microbial Community, Diversity Structure, and Predictive Functional Profiling in Continuous Cucumber Planted Soil Affected by Diverse Cropping Systems in an Intensive Greenhouse Region of Northern China
Source: Int J Mol Sci. 2019 May 28;20(11):2619. doi: 10.3390/ijms20112619 (PMC6600451; doi:10.3390/ijms20112619)
Supplement: Supplementary file 1 [file ijms-20-02619-s001.zip › Supplimentry File-1.docx]

**Title: HiSeq base molecular characterization of soil microbial community, diversity structure and predictive functional profiling in cucumber planted soil affected by diverse cropping system in an intensive greenhouse region of Northern China.**

**.**

Supplementary data includes;

Tables

Figure

| **Sample Name** | **Raw Tags** | **Clean Tags** | **Effective Tags** | **Max. length** | **Min length** | **Effective Ratio (%)** |
| --- | --- | --- | --- | --- | --- | --- |
| FC1 | 64757 | 64640 | 61068 | 489 | 392 | 93.31 |
| FC2 | 68496 | 68357 | 64687 | 489 | 306 | 93.37 |
| FC3 | 59210 | 59090 | 56211 | 489 | 343 | 93.89 |
| SC1 | 74194 | 74059 | 69760 | 489 | 350 | 94.10 |
| SC2 | 61711 | 61589 | 58678 | 487 | 342 | 94.16 |
| SC3 | 62073 | 61964 | 58205 | 489 | 320 | 93.90 |
| CC1 | 66603 | 66464 | 62288 | 489 | 320 | 93.86 |
| CC2 | 70707 | 70562 | 66654 | 489 | 361 | 94.15 |
| CC3 | 60559 | 60451 | 57665 | 489 | 320 | 94.53 |
| NCCC1 | 69038 | 68900 | 65089 | 489 | 314 | 92.85 |
| NCCC2 | 58811 | 58696 | 55517 | 489 | 365 | 92.47 |
| NCCC3 | 60628 | 60518 | 57260 | 489 | 418 | 93.01 |
| LLC1 | 55371 | 55245 | 53462 | 489 | 397 | 94.34 |
| LLC2 | 59697 | 59587 | 57572 | 489 | 316 | 95.76 |
| LLC3 | 59509 | 59386 | 56935 | 489 | 316 | 93.45 |

Table S1. Description of the all identified sequences affiliated with soil samples using 16S rDNA Illumina HiSeq analysis after experiments in 2017.

| **Sample Name** | **Domain** | **Phylum** | **Class** | **Order** | **Family** | **Genus** | **Species** |
| --- | --- | --- | --- | --- | --- | --- | --- |
| FC1 | 52935 | 52553 | 49992 | 45395 | 37170 | 20053 | 1452 |
| FC2 | 57065 | 56624 | 53709 | 48339 | 39422 | 19606 | 1019 |
| FC3 | 50027 | 49324 | 46714 | 41867 | 34333 | 18389 | 1082 |
| SC1 | 60891 | 60474 | 57734 | 52622 | 42669 | 22260 | 1666 |
| SC2 | 52715 | 52109 | 49826 | 45401 | 38247 | 20487 | 1165 |
| SC3 | 51405 | 50964 | 48574 | 44054 | 36240 | 19194 | 1076 |
| CC1 | 54187 | 53716 | 51262 | 46119 | 38124 | 19719 | 1870 |
| CC2 | 59373 | 58886 | 56230 | 51499 | 42960 | 23400 | 1605 |
| CC3 | 52270 | 51716 | 49449 | 44957 | 38243 | 21368 | 1448 |
| NCCC1 | 57980 | 57537 | 54803 | 50042 | 42028 | 23307 | 1523 |
| NCCC2 | 49653 | 49308 | 47192 | 43676 | 37330 | 21157 | 1483 |
| NCCC3 | 50944 | 50513 | 48391 | 44564 | 37084 | 20535 | 1506 |
| LLC1 | 49403 | 49066 | 47098 | 43368 | 36196 | 20845 | 1410 |
| LLC2 | 53098 | 52657 | 50233 | 45894 | 38448 | 21122 | 1809 |
| LLC3  Table S2. Species taxonomy analysis affiliated with soil samples using Illumina HiSeq analysis after experiments in 2017. The classification of microbial species is generally divided into seven levels: Domain, phylum, class, order, family, genus and species, and each OTU represents a set of classification levels for a certain type. | 51769 | 51383 | 48935 | 44634 | 37122 | 20314 | 1327 |


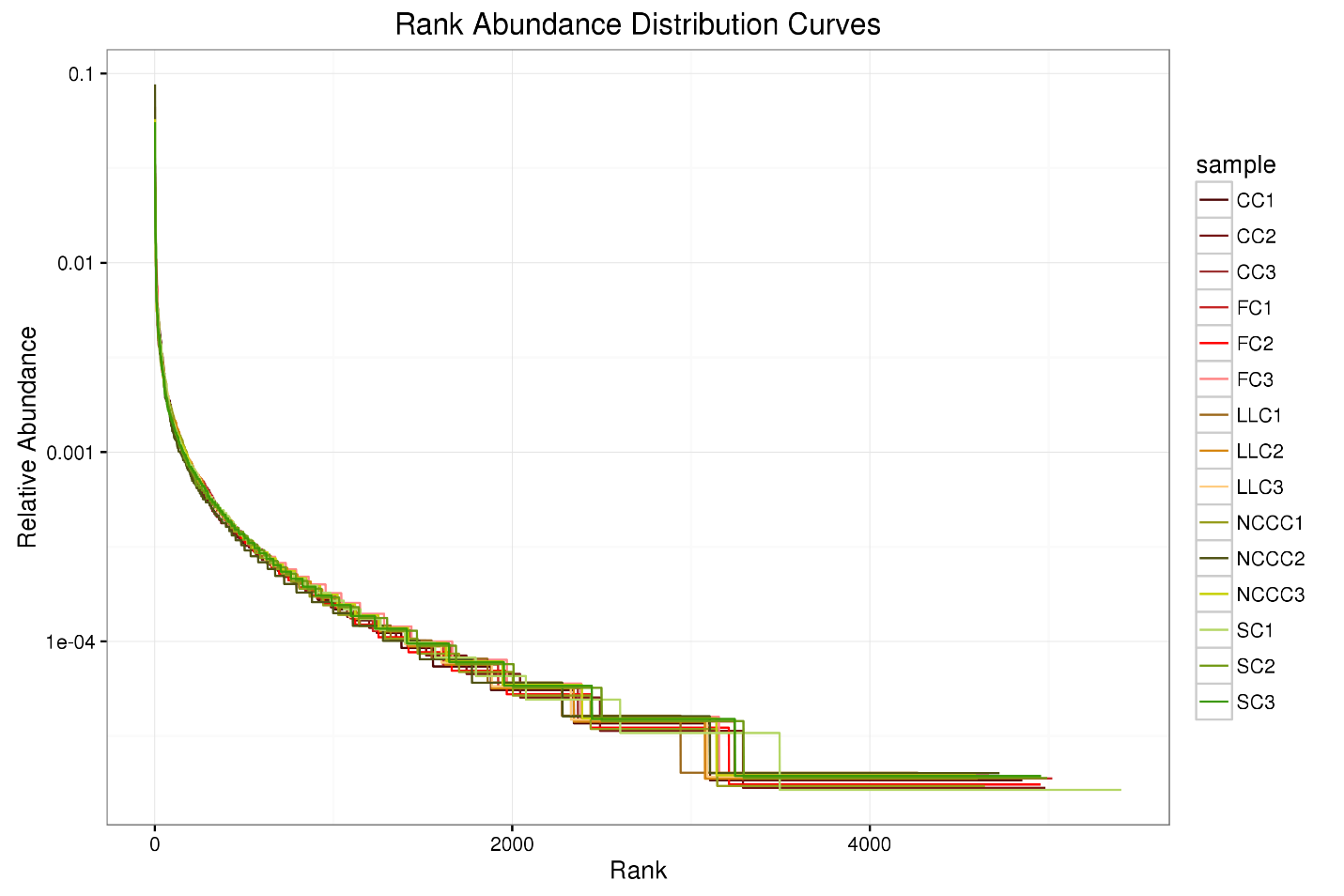


Fig. S2: Rank Abundance curve of different treatments.
